# Improving Risk Assessment in the European Food Safety Authority: Lessons From the European Medicines Agency

**DOI:** 10.3389/fpls.2020.00349

**Published:** 2020-04-06

**Authors:** Sevasti Chatzopoulou, Nélida Leiva Eriksson, Dennis Eriksson

**Affiliations:** ^1^Department of Social Sciences and Business, Roskilde University, Roskilde, Denmark; ^2^Department of Chemistry, Lund University, Lund, Sweden; ^3^Department of Plant Breeding, Swedish University of Agricultural Sciences, Alnarp, Sweden

**Keywords:** risk, assessment, food, regulatory, governance

## Abstract

The recent Regulation (EU) 2019/1381, published on the 6th September 2019, aims to improve the transparency and sustainability of the EU risk assessment in the food chain by amending the General Food Law Regulation (EC 178/2002) and a number of other regulations related to the food sector. This Regulation is introduced as a response to the Fitness Check of the General Food Law Regulation as well as a response to public concerns expressed by a European Citizens’ Initiative on glyphosate and pesticides. This article evaluates the amendments introduced by Regulation 2019/1381with respect to the institutional and regulatory environment in the food chain and more specifically concerning the risk assessment procedure. For this purpose, we perform a comparison of the institutional and organizational characteristics of the European Food Safety Authority (EFSA) and European Medicines Agency (EMA) in relation to the processes of risk assessment and risk evaluation, especially the processes surrounding genetically modified foods and pesticides, and how these characteristics affect the politicization of these processes. We conclude that the risk assessment process followed by EFSA would have benefitted and become more effective and less politicized, if the recent Regulation 2019/1381 had introduced some of EMA’s institutional structures and methods on risk evaluation.

## Introduction

In the past, food policy was within the competence of the Member States in the European Union (EU). Following a series of crises in the late 1990s [the Bovine Spongiform Encephalitis (BSE) crisis, *E. coli* etc.], the Member States transferred the food policy competences, particularly with respect to food safety, to the EU institutions in the early 2000s. This transfer resulted in institutional and legislative changes and introduced a number of Regulations, Directives and decisions. The Prodi Commission (1999–2003) created the Directorate General SANTE^1^ and the General Food Law (GFL, Regulation 178/2002) established the European Food Safety Authority (EFSA) (Chatzopoulou,2019b). These developments reflected the need for an integrated policy at the supranational level that could lead to harmonization of food and feed safety rules and marked the Europeanization of food policy across the EU Member States (Alemanno, 2006; Chatzopoulou, 2015, 2019a). European people received this food policy transition mostly positively (Eurobarometer^2^, 2019, p. 28). However, EFSA has been criticized for lack of effectiveness particularly with respect to risk analysis, selection of data and information, risk communication; lack of transparency and broad representation of the available scientific knowledge (Chatzopoulou, 2015); and the long duration risk assessment processes^3^, which also created delays in the subsequent authorization of applications (EuropaBio, 2016; Wesseler and Kalaitzandonakes, 2019). These concerns about transparency resulted in some degree of public dissatisfaction and contestation of EFSA’s work. Moreover, concerns about conflict of interest among EFSA’s experts led the European Parliament to withhold EFSA’s budget resulting in stricter administrative rules (Chatzopoulou, 2015).

Following the adoption of the European Citizens’ Initiative (ECI) Regulation in 2010^4^, but before it entered into force, Greenpeace claimed to have collected 1 million signatures calling for a moratorium on genetically modified (GM) crops. During 2014–2018, the Commission launched the Fitness Check of the GFL Regulation, which also identified various concerns regarding the risk assessment of GM organisms (GMOs) and the governance of EFSA. In addition, following a series of critiques by various non-governmental organizations, an ECI, that had collected 1,070,865 signatories^5^, to “Ban Glyphosate and Protect People and the Environment from Toxic Pesticides”^6^ was presented to the Commission on the 23^rd^ of October 2017. This initiative raised concerns on the transparency and sustainability of EFSA’s risk assessment processes in the food chain. A public hearing was organized at the Parliament on 20 November 2017^7^. Responding to these concerns, the European Commission (EC) submitted on 11 April 2018 a regulation proposal to the Council and the European Parliament (EP) that led to Regulation 2019/1381. This Regulation introduced amendments in Regulation (EC 178/2002) on general food law and a number of Regulations related to GM food and feed (1829/2003) and feed additives (1831/2003) along with eight legislative acts dealing with specific sectors^8^ in the food chain^9^. Thus, the paper focuses mainly on the regulated products GM and pesticides and not on non-regulated ones (e.g., contaminants). The amendments aim to improve the transparency, reliability and independence of studies submitted to EFSA in order to support EFSA’s risk assessment process.

The Regulation emphasizes the proactive and automatic communication to the public, at an early stage of the risk assessment, of all studies submitted to EFSA for risk assessments via EFSA’s website thereby strengthening the transparency and underpinning EFSA’s assessments while protecting legitimate confidential business information^10^. Moreover, the Regulation introduces a greater involvement of the Member States in the Management Board in line with the inter-institutional “Common Approach on EU decentralized agencies^11^,” as it is in the case of the European Medicines Agency (EMA). For instance, the Member States are encouraged to be active in the nomination of scientific panel experts for risk assessment. Such a change is expected to broaden the number and type of experts with respect to disciplines and geographical distribution.

Taking stock on the existing literature on risk governance, this paper addresses the overall question: *to what extent will the recent Regulation, on the transparency and sustainability of the EU risk assessment in the food chain, improve the risk assessment process in EFSA and increase trust among the European people?*

To address this question, this paper links the amendments made by the Regulation, in the governance of risk analysis for EFSA, to the corresponding ones in EMA. This comparison is relevant in understanding the governance of risk assessment because EFSA and EMA belong to the same cluster of agencies, both are under DG SANTE and their areas of expertize are connected, both agencies consider aspects of health and environment and perform risk assessment for products that will be introduced to the market^12^. The foundation of both agencies, aimed to ensure that risk assessment processes, are based on objective scientific knowledge. However, the governance of risk assessment of food and feed biotechnology in EFSA has been highly contested, in comparison to medical biotechnology in EMA. This contestation is also accompanied by a low acceptance of food and feed biotechnology. For example, the acceptance of genetic modification by society differs among the two sectors, e.g., food and agriculture and health and medicine (Olynk Widmar et al., 2017).

The paper is structured as follows: After presenting the competences and governance structures of EFSA and EMA, the following section discusses the real problem that raises criticisms, over time, in relation to the governance of risk assessment in EFSA. A description of the policy process is also presented, followed by the discussion of the results.

## The Main Critical Issue: Backdrops of Risk Management Process

The founding of EFSA and EMA aimed to support the Commission’s work by providing scientific based opinions based on risk assessment and risk evaluation processes respectively. However, EFSA’s risk assessment opinions on food and feed biotechnology have been criticized, especially with respect to transparency (publication of studies used for the assessment^13^) and demonstrated politicization elements (Löfstedt, 2004; Chatzopoulou, 2015). EMA, on the other side, does not face such critiques concerning biomedicine and genetic medicine. This article suggests that this difference is related to the dissimilar governance of risk assessment in these two agencies, implicating directly the risk management processes. One contrasting difference is the role of the member states in the risk assessment process. The member states’ involvement matters as it shapes, as it would be expected, the governments’ attitudes and possibly also the public opinion. Regulatory systems and *ad hoc* decisions are not only a response to public attitudes but they also contribute to forming public attitudes in a significant way (Qaim, 2016, p. 117).

Olynk Widmar et al. (2017) demonstrate that GM acceptance in the society differs among sectors, e.g., it depends on if GM food and feed are used and associated with human health or with plant biotechnology. These studies show that GMOs used in pharmaceutical production do not face the same contestation as GMOs used directly for food or food processing (Qaim, 2016). For example, a series of scientific controversies among member states created delays during the risk management processes in the case of maize^14^ (Qaim, 2016; Eriksson and Chatzopoulou, 2017). Following the risk assessment process by EFSA, the disagreements emerge in the comitology that consists of civil servants from all Member States and oversees the Commission’s use of delegated powers. When qualified majority voting (QMV) cannot be reached in this committee, then the Appeal Committee can overrule the Commission by QMV. Most often, the Appeal Committee ends up with no decisions and then the Commission has the final responsibility^15^ (Christiansen, 2019, p. 111). In the great majority of cases, this results in (1) a favorable scientific opinion by EFSA, (2) no opinion through comitology, or (3) threats of court cases of inaction (ibid). In the case of medicines registration by EMA, there is no comitology procedure. Formally, the Commission’s decision is based on EFSA’s risk assessment opinion. And although the Commission has authorized GMO applications in the past years for food and feed use, these decisions were not for cultivation. *If the decision would have only been based on EFSA’s risk assessment*, several additional GM products for food and feed, or also for cultivation *might* have been approved by the EC. Consequently, it can be argued that in practice there is still a moratorium on the approval of GMOs for cultivation, as the only GM crop that has been authorized for cultivation in recent years (the Amflora potato) had its authorization annulled in 2013. The EU Court argued that there was a procedural error in the approval process (General Court of the European Union, 2013) due to insufficient involvement of the Member States in the standing committee by the Commission. In other words, the final rejection of the Amflora potato was based on national politics and interests and not on EFSA’s science based risk assessment. Although the EU introduced an opt-out mechanism (Directive EU 2015/412) in 2015 which allowed member states to restrict or prohibit cultivation of authorized GM crops in their territory, this did not resolve these issues (Eriksson et al., 2019).

Such incidents reflect a broader uncertainty with respect to the risk management at the Commission level (following EFSA’s risk assessment), which the existing decision-making and governance processes have not been able to address adequately. These incidents also demonstrate the importance of the national views and interests in the decision-making, which seem not to be based on EFSA’s scientific risk assessment on safety for health and the environment (Qaim, 2016, p. 116). Thus, politicization is leading to outcomes that are not based on scientific knowledge, affecting the legitimacy and reputation of the governance of risk assessment processes and the role of EU institutions, namely EFSA.

In light of these arguments, this paper analyses to what extent the current development with the Regulation 2019/1381 will be able to address effectively issues on the governance of EU risk management. For this purpose, we compare the institutional and organizational characteristics of EFSA and EMA with respect to the risk assessment process, which constitutes the basis of risk management by the Commission. This comparison is expected to allow us to unfold and understand the necessary changes in the EU institutional and regulatory environment with respect to risk assessment and the risk management procedure on food biotechnology. Such changes can potentially contribute to elimination of delays and promote innovation in food biotechnology in a responsible manner.

## The Institutional Structures and Risk Assessment in EFSA and EMA

Despite their similarities, the two agencies, EFSA and EMA, follow different governance structures, which affect their functioning, reputation, and legitimacy. Governance provisions and structures determine the control mechanisms used by the agencies’ principals (the Commission and the Member States). EFSA is the most recently founded of the two agencies, and as above mentioned, it was established in 2002. Furthermore, while the risk evaluation of medicines has been harmonized more at the global level, in the case of certain food aspects, such as GMOs, there is significant divergence concerning risk assessment processes (e.g., process versus product based)^16^. EFSA’s establishment aimed to provide independent scientific advice and clear communication on existing and emerging risks in the area of food and feed safety, animal health and welfare as well as plant health (European Food Safety Authority, 2014).

The GFL (Regulation 178/2002)^17^ defined the rules for the entry of new food and/or feed products into the EU market, established EFSA, and set out certain procedures related to food safety. The GFL provides four measures: (1) the establishment of the Rapid Alert System for Food and Feed (RASFF), (2) the Standing Committee on Plants, Animals, Food and Feed (PAFF), (3) the adoption of emergency measures, and (4) the establishment of a general plan for crisis management. In addition, it includes three inter-related components of risk analysis: risk assessment, risk management and risk communication (Box 1).

**Box 1. The three interconnected components of risk analysis according to the EU General Food Law, Regulation 178/2002 (L31/7, L31/8).**

9. “Risk” means a function of the probability of an adverse health effect and the severity of that effect, consequential to a hazard;
10. “Risk analysis” means a process consisting of three interconnected components: risk assessment, risk management and risk communication;
11. “Risk assessment” means a scientifically based process consisting of four steps: hazard identification, hazard characterization, exposure assessment and risk characterization;
  The risk assessment must be undertaken in an independent, objective and transparent manner based on the best available science.
12. “Risk management” means the process, distinct from risk assessment, of weighing policy alternatives in consultation with interested parties, considering risk assessment and other legitimate factors, and, if need be, selecting appropriate prevention and control options; (Regulation 178/2002:7.
13. “Risk communication” means the interactive exchange of information and opinions throughout the risk analysis process as regards hazards and risks, risk-related factors and risk perceptions, among risk assessors, risk managers, consumers, feed and food businesses, the academic community and other interested parties, including the explanation of risk assessment findings and the basis of risk management decisions;’ (Regulation 178/2002:8).

The GFL also defined the principles of EFSA governance. Taking into account the opinion of the EP, the Commission proposes EFSA’s 14-member Management Board. The selection is based on the members’ experience and expertize and not on nationality (European Food Safety Authority, 2011), but it should secure the broadest possible geographic distribution within the Union (Reg. 178/2002, Art. 25). This process constituted an innovation in the EU agencies’ governance, since until then territorial representation in the agencies Management Board was important. Additionally, four of the Management Board members should represent organizations such as consumers and other interests in the food chain (European Food Safety Authority, 2011).

The Management Board appoints an Executive Director who is responsible for the implementation of the financial rules of the Authority and has to ensure the adequate organization of the legality of transactions (European Court of Auditors, 2011). The Executive Director also has the responsibility for the day-to-day management of EFSA and is supported by the Heads of department, Heads of unit, the Chief Scientist and the Senior Policy Adviser^18^. A Scientific Committee and 10 Scientific Panels (corresponding to different policy areas^19^) and their working groups supports EFSA’s risk assessment work. According to the existing legislation, the Scientific Committee^20^ and the scientific panels provide the Authority’s scientific opinions to the Commission, each within their own spheres of competence (Commission Regulation (EU), 2017). The Scientific Committee consists of the chairs of the Scientific Panels complemented by six independent scientific experts who do not belong to any of the Scientific Panels and focuses on the coordination and consistency of the scientific opinion procedure (Reg. 178/2002). The scientific panels are composed of independent scientific experts who carry out scientific assessments, organize public hearings where necessary, and develop related assessment methodologies. These are appointed for 3-year periods, similar to the ones in EMA. However, they do not secure the geographic representation of all member states as it occurs in EMA’s scientific committees.

When EFSA receives a market application first validates its completeness or if it needs more information to proceed. Then EFSA’s relevant Panel establishes a working group that develops a draft and submits it to the Panel for discussion and often to public consultations. For example, for the GMO Panel, there are three permanent working groups: molecular data, food and feed, and environmental risks). The working group consists of members of the relevant Panel and a number of additional scientists from specialist fields. This working group assesses the available scientific information from the Member States, research institutes or companies. EFSA may request more data directly from the applicant. An important aspect is the defining of a timetable of the process from the beginning, which depends on each case. The adoption of the assessment, usually a scientific opinion (it can also be a Statement, Guidance Document or another type of output), is based on majority in the relevant Panel at a plenary meeting^21^.

Based on EFSA’s risk assessment, the Standing Committee on Plants, Animals, Food and Feed (PAFF)^22^ under the Commission decides the final authorization of the product. The PAFF is an intergovernmental committee composed of representatives of all Member States and is chaired by a European Commission representative that also exemplifies the networked characterization of the EU agencification^23^. Its mandate covers the entire food supply chain – from animal health issues on the farm to the product on the consumer’s table. The PAFF plays a key role ensuring that the EU measures on food and feed safety, animal health and welfare, and plant health are practical and effective. The PAFF delivers opinions on draft measures that the Commission, who is responsible for the risk management, intends to adopt. This is the first committee where all the member states are represented in the risk analysis process. But, this Committee constitutes part of the risk management not the risk assessment (scientific level) and constitutes a significant difference when compared to EMA.

Similarly to EFSA, EMA consists of scientific committees, seven of them, and a number of working parties and related groups, which conduct the scientific work. The EU pharmaceutical legislation was introduced in 1965 as a reaction to the thalidomide scandal (malformation effects on babies by the medicine for pregnant women). EMA was founded in 1993 by merging the pre-existing Committee for Human Medicinal Products (CHMP) former CPMP (Committee for Proprietary Medicinal Products) and the Committee for Veterinary Medicinal Products (CVMP). This merger initially created the European Agency for the Evaluation of Medicinal Products (EMEA) that was renamed as European Medicines Agency (EMA) in 2004^24^. EMA’s was expected to further the efficient and flexible implementation of EU legislation on pharmaceuticals, and ensure rapid access of new products to the Community market (Sauer, 1996, p. 23, as cited Groenleer, 2009, p. 145). In order for a medicinal product to be placed on the EU market, it has to follow the core pharmaceutical regulation, namely the marketing authorization requirement. There are three different procedures for authorizing medicines: the centralized procedure, the mutual recognition/decentralized procedure and the national procedure (Wirtz, 2017). For certain biotechnology-derived and high tech products the centralized procedure is mandatory. While the marketing authorization is granted by the EU Commission, the scientific assessment of the application is carried out by the EMA.

Each of the EMA committees follows its own rules of procedure. Each committee appoints a rapporteur who prepares an assessment report, which the committee will consider and eventually adopt as part of a scientific opinion or recommendation. For certain procedures, a “co-rapporteur” also prepares an assessment independently from the rapporteur^25^. The work of the rapporteur and co-rapporteur is supported with resources and expertize by an assessment team with necessary expertize and resources. In addition, the EMA secretariat provides technical, scientific and administrative support for each assessment. In order to mobilize the best expertize for medicines evaluation, regardless of where experts are geographically based, rapporteurs and co-rapporteurs can establish multinational assessment teams by including experts from other Member States as well as their own. The EMA committees try to reach their conclusions by consensus whenever possible, but if not the committee holds a vote, which follows specific procedures and rules^26^. For this purpose, Member States have to liaise with the Management Board and the EC in order to ensure that the final composition of the Committees covers the scientific areas relevant to its tasks. The committee considers the final assessment report and eventually adopts it as part of a scientific opinion or recommendation.

Since 2004, CHMP carries out the assessment and evaluation. This Committee often consults the Pharmacovigilance Risk Assessment Committee (PRAC) in relation to risk assessment. Directive 2001/83/EC and Regulation (EC) No 726/2004 lay down specific rules concerning the pharmacovigilance of medicinal products for human use and set up the PRAC. Accordingly, the PRAC is responsible for the risk management of the use of medicinal products for human use including detection, assessment, minimization and communication related to the risk of adverse reactions, design and evaluation of post-authorization safety studies and pharmacovigilance audit.

Both the CHMO and PRAC committees consist of a Chair and one member and one alternate member appointed by each of the EU Member States and one member and one alternate member appointed by each of the EEA-EFTA States. The EC also appoints several representatives. Two experts (one member and an alternate) on clinical pharmacology and pharmaco-epidemiology to ensure that the relevant expertize is available within PRAC, other two (one member and one alternate), to represent healthcare professionals; and finally two more (one member and one alternate) to represent patient organizations. All members, except those appointed by EU and EEA-EFTA states, are appointed based on a public call for expressions of interest and after consulting the EP based on their relevant expertize in pharmacovigilance matters and risk assessment of medicinal products for human use. The members and the alternates of the Committees are appointed for a term of 3 years, which may be prolonged once. The aim is to guarantee the highest levels of specialist qualifications and a broad spectrum of relevant expertize. Since all Member States are involved in the risk evaluation as part of the scientific committees for pharmaceuticals, scientific differences in national opinions are resolved before the EMA provides its scientific opinion to the Commission, who then mostly rubberstamps EMA’s opinions.

When concerns over the safety or benefit-risk balance of a medicine or class of medicines are raised, a referral procedure, which can be started by the EC, a Member State or the company that markets the medicine, is used to resolve such issues. In a referral, the medicine, or class of medicines, is “referred” to EMA so that it can conduct a scientific assessment on behalf of the EU and then make a recommendation for harmonized position across the EU. There are a number of reasons why a referral may be started, ranging from concerns over the safety to disagreements among Member States on the use of the medicine. Safety-related referrals are assessed by the PRAC and then either by the CHMP or, for nationally authorized medicines, by the Coordination Group for Mutual Recognition and Decentralized Procedures-Human (CMDh). All other referrals on human medicines are assessed by the CHMP only. For most referrals, the EC issues a decision to all Member States reflecting the measures to take to implement the Agency’s recommendation^27^.

## Chronology of Events

**FIGURE 1:**
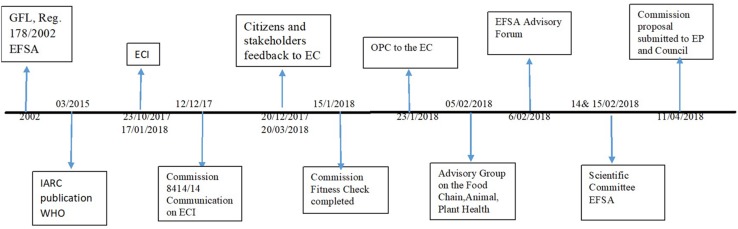
Timeline of events in relation to the Commission’s legislative proposal for GFL until the EC submitted its proposal to EP and Counsil.

Figure 1 presents a chronological record of events since the GFL founding in 2002 until the submission of the legislative proposal by the Commission to the EP and the Council in April 2018. In December 2010, Greenpeace and Avaaz submitted a pilot ECI with one million signatures in accordance with the rules established by the Lisbon Treaty in 2009. This first pilot ECI responded to the first authorization in 12 years by the Commission in March 2010, for the cultivation of a GM crop in Europe^28^. The 2010 ECI called for a moratorium on all new authorizations and a review of the GM approval process, claiming that the existing authorization raised serious health and environmental concerns. As the ECI process was not formally implemented at this point, the EU institutions did not have to take action. Years later, in March 2015, in disagreement with EFSA’s scientific opinion, the International Association for Research Cancer (WHO, IARC) “classified glyphosate as ‘probably carcinogenic to humans’ (Group 2A),” which triggered a lot of concern about the objectivity of science in the society. Between 23/10/2017 until 17/01/2018, a most recent ECI “Ban glyphosate and protect people and the environment from toxic pesticides” was launched, which indicated concerns on the transparency in the risk management process by EFSA. This recent ECI became one of the four successful ECIs since the Regulation (EU) No 211/2011 Article 11(4) of the Treaty of the European Union was put into practice in 2012^29^ and the Commission responded according to the Lisbon Treaty rules.

Furthermore, during 2014–2018, the Commission launched a Fitness Check in order to address if the existing GFL is still “fit for purpose” regarding its relevance and effectiveness, efficiency, coherence, and whether it should be simplified so that it can become less costly. The Fitness Check recognized the positive outcomes of the EU food and feed safety policy, but it also acknowledged that there is space for improvement in “the implementation of the functional separation of the risk assessment and risk management at EU level, set out in the GFL Regulation”^30^. In addition, the Commission received feedback 20/12/2017-17/01/2018 and started an Open Consultation 23/1/2018-20/3/2018. Moreover, this was discussed at various fora with different actors, namely the Advisory Group on the Food Chain and Animal and Plant Health^31^; the EFSA Advisory Forum (national food safety authorities on 6th February 2018); the Commission Expert Group on General Food Law^32^ (5th March, 2018) and finally the Scientific Committee of EFSA^33^ (14 and 15/02/2018). This demonstrates a long process that involved a variety of public and private actors, before the Commission formulated its legislative proposal on 11/04/2018.

## Changes Introduced by the Recent Commission Regulation 2019/1381 on the Transparency and Sustainability of the EU Risk Assessment in the Food Chain

On 11 April 2018, the Commission submitted a proposal to the Council and the Parliament for a regulation on the transparency and sustainability of the EU risk assessment in the food chain. This proposal regulation aimed to amend the GFL Regulation (EC 178/2002) and a number of other regulations related to, amongst others, GM crop cultivation, food and feed uses (1829/2003), and food and feed additives (1831/2003). The recent Reg. 2019/1381 addresses aspects of governance and by introducing a change in the composition of the Management Board, in a way, recognizing the importance of the representation of all member states, as it is the case in EMA:

“It is thus appropriate to include representatives of all Member States of the European Parliament and of the Commission as well as of civil society and industry organizations in the Management Board, while providing that those representatives should have experience and expertize not only in the fields of food chain law and policy, including risk assessment, but also in the fields of managerial, administrative, financial and legal matters and ensuring that they act independently in the public interest” (Reg. 2019/1381, Art.14, L231/3).

Responding to the shortcomings in the Authority’s high level expertize identified by the Fitness Check (Art. 16, L231/3), the new Regulation emphasizes the importance of greater involvement of the Member States in the Management Board by nominating scientific panel experts for risk assessment. This change would be more in line with the inter-institutional Common Approach on Union Decentralized Agencies in the effort to increase the consistency of the EU agencies’ management board model. Such a change is expected to broaden the number and type of experts with respect to disciplines, number, and geographical distribution. In order to do so, it is suggested to provide better financial compensation, which is currently considered low, in order to attract highly qualified experts. However, expansion of the Management Board (Reg. 2019/1381) and the number of candidates does not adequately address the problems in the actual structure of the risk assessment process, which is directly linked to the expertize of the Authority’s scientific panels. Neither, “a more active role to ensure that a sufficient pool of experts is available to meet the needs of the Union risk assessment system” of the Management Board or the member states in the appointment of the scientific panels’ members would be sufficient (Reg. 2019/1381). This change does not describe precisely what is an “active role” and how this could ensure “high level of scientific expertize, independence and multidisciplinary expertize.” While the national scientific organizations are involved “in drafting preparatory scientific opinions to be peer-reviewed and adopted” (Art. 18) by the scientific panels they are not represented in the preparation phase (ibid).

Regulation 2019/1381 focuses significantly on risk communication through: (1) Automatic publication of all studies and supporting information submitted to EFSA for risk assessment, in an electronic format that would be publicly available and easily accessible; (2) Stakeholders would be consulted on submitted studies, and confidentiality would be protected in justified circumstances; (3) A specific procedure would be implemented for renewals of substances already authorized; and (4) The Commission would, via delegated act, adopt a general plan for risk communications in the agrifood chain (Comitology Newsletter #52, 2018). While this is important, it raises concerns in the industry concerning confidentiality and property rights with implications on research and innovation in the sector^34^. Most importantly, these changes do not tackle the identified concerns on the risk assessment process adequately as they concentrate on the risk communication.

When the EP received the proposal from the Commission, the Special Committee on the EU authorization procedure for pesticides (PEST) was established and held 12 meetings during 2018. In the EP the Environment, Public Health and Food Safety Committee (ENVI) has been assigned the responsibility to write the report. The first Rapporteur was Renate Sommer (EPP, DE), who suggested in its draft report that the EP would prefer to align the EFSA rules with those of other EU agencies (e.g., the EMA) as much as possible, but ensure that confidential information does not become available at the time the application is submitted but when EFSA adopts its final opinion^35^. Early publication of information could jeopardize innovation and jobs creation as the industry would be reluctant to invest in EU countries. Renate Sommer resigned^36^ in protest at the final shape of her report, when the plenary voted by 427 in favor (172 against, 67 abstentions) of amendments^37^ to the draft EFSA reform on 11 December 2018. Mrs Sommer characterized the decision a “populist” move that will harm innovation and “endanger the whole food chain.” The Spanish MEP Pilar Ayuso González took over the representation in trilogues, despite her vote against the final EP report. The Council reached an internal position in December.

**FIGURE 2:**
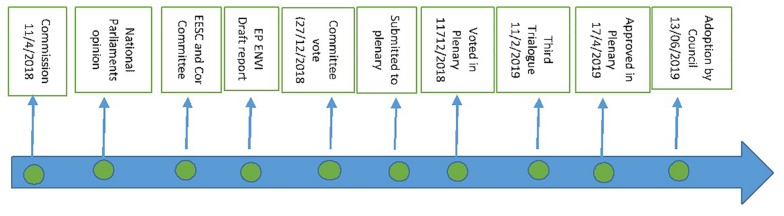
Procedure on Commission’s legislative proposal after it was submitted to EP and Council.

During the process, there were many disagreements in the Council. For example, the Dutch government criticized several elements, in particular the notion of granting EFSA more funds to fulfill the required extra tasks. Figure 2 presents a recount of events since the submission of the Commission’s proposal to the Council and the EP^38^ until the adoption of a new regulation on the transparency and sustainability of the EU risk assessment in the food chain by the Council on 13 June 2019. The Parliament finalized its position by a vote and agreement in the Plenary (11/12/2018) followed by the adoption by the Council (12/12/2018). A provisional agreement was reached at the third trilogue meeting (11/02/2019), and was endorsed in the ENVI committee (20/02/2019). “The provisional agreement sets out that: supporting data and information linked to an application for authorization will be made public by the EFSA after the assessment of the validity of the application unless the applicant proves that this could significantly harm its interest and requests confidential treatment by EFSA. The applicant will be able to file a confirmatory request if s/he disagrees with EFSA’s assessment of confidentiality. In this case, the information cannot be made public until a final word is said. The Commission will be able to request EFSA to commission its own verification studies in exceptional controversial cases of high importance for the society and member states will have a more active role in helping EFSA attract more and the best scientists to participate in Scientific Panels. Risk communication among all actors – the Commission, EFSA, member states and public stakeholders – will be improved to ensure a more coherent, transparent and continuous flow of information throughout the risk assessment process^39^.” The Parliament approved the agreement (17/04/2019) and the Council has formally adopted a new Regulation^40^ on the transparency and sustainability of the EU risk assessment in the food chain on June 13, 2019 based on the Commission’s proposal^41^.

The new Regulation amends the GFL Regulation and eight legislative acts dealing with specific sectors of the food chain: GMOs (cultivation and for Food/Feed uses), feed additives, smoke flavorings, food contact materials, food additives, food enzymes and flavorings, plant protection products and novel foods^42^. Following its entry into force 20 days after publication, September 6, 2019, it will become applicable 18 months later thus by the end of March 2021. The Regulation introduced one important change with respect to the role of the Member States in the governance of EFSA. When the Regulation will apply, each Member State will nominate a representative to the Management Board, increasing their role and level of responsibility in supporting EFSA and ensuring an increased scientific cooperation. The selection of the Member States’ representatives in the new Management Board will be based on specific requirements such as relevant experience and expertize in the field of the food chain legislation and policy, including risk assessment. The strict criteria of independence will also have to be fulfilled (ibid).

## Discussion

The EU divides the feed and food risk analysis in risk assessment, risk management, and risk communication, where EFSA is responsible for risk assessment, the Commission for risk management and they share risk communication depending if it is an assessment or management issue. This division was a response to the mismanagement of the BSE crisis and the high degree of politicization on food policy, which is today reflected in the rationale behind the governance of EFSA where both the Commission and the Member States instated a “police patrol” type of control. However, this division of competences did not decrease the politicization; at the contrary, it complicated the process by distinguishing the two different levels, one scientific (EFSA) and one political (EC). The scientific committees of EFSA have been criticized for not representing broadly the available scientific knowledge. The agency’s work depends on its capacity to combine expertize from the Member States. National scientific organizations contribute to EFSA’s work through their participation as experts to EFSA’s scientific panels, and by providing EFSA with scientific data and studies.

**FIGURE 3:**
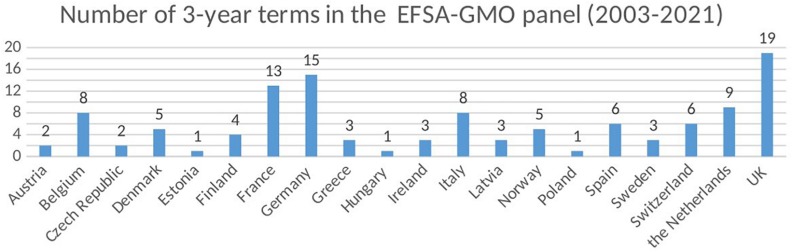
Composition of the EFSA-GMO panels by the EU member states from 2003 to 2021.

However, the representation of all Member States in the scientific panels is not required. This was not changed by the recent amendments by Regulation 2019/1381. As a result, only a small number of Member States (six) provide more than two thirds of the experts on EFSA’s ten scientific panels that can have maximum 21 members^43^. In the last round that started in June 1st, 2018 6 member states (France, Germany, Italy, Netherlands, Spain, United Kingdom) provide 109 out of the 168 experts in the 10 scientific panels of EFSA. Some countries have no representative at any panel, and there are increasing difficulties in attracting enough new candidates to work in them. Here the first difference from EMA that has more financial and human resources and all Member States are represented in the scientific committees. Consequently, the risk assessment process in EFSA by the independent scientists of the scientific committees does not involve all Member States. In praxis, the Member states’ different views and interests are expressed and negotiated during the voting in the Standing Committee on the Food Chain and Animal Health under the EC. As a result, although the scientific opinion provided by EFSA constitutes the point of departure for the decisions on the authorization of food and feed, these decisions are strongly affected by national politics and views. When it comes to a highly contentious field such as GMOs and their derived products, the PAFF almost never reach a common decision. There are always a number of EU member states that vote against authorization, despite a favorable scientific recommendation by EFSA (Smart et al., 2015). Looking at the composition specifically of the EFSA GMO panel over the years since its inception, it is obvious that there is a lack of representation from several countries. The panel has had 16–21 experts appointed for 3-year periods. In total, over the periods since 2003 and until the most recent (2018–2021), there has been an accumulated 117 appointments and 72% of these come from only eight countries (Figure 3). When this is compared to the voting behavior of these countries in 2003–2015, five of them (Belgium, Germany, the Netherlands, Spain, and United Kingdom) are characterized by a strong inclination to vote in favor of authorization and thus following EFSA’s scientific recommendation. Two of them (France and Italy) have a tendency to abstain from voting and occasionally vote either for or against authorization. Several countries that tend to always vote against authorization of GMOs have never been represented in the EFSA GMO panel, such as Croatia, Cyprus, Lithuania and Luxembourg, or represented very few times, such as Austria, Hungary and Poland.

It may be argued that EU Member countries without representation can still be active during the decision making process by submitting their comments and then getting a point-by-point reply afterward in annex to EFSA’s opinion. However, the possibility of giving comments does not really compensate for their lack of representation. In fact, this has only created delays, instead of contributing to effectiveness, as countries lacking representation tend to present their own scientific evidence at late stages making the process to start again. In contrast, differences in national opinions regarding the approval of pharmaceutical products are resolved before the EMA provides its scientific opinion to the EC given that all Member States are involved in the risk evaluation as part of EMA’s scientific committees.

Any differences in national scientific opinions regarding the approval of pharmaceutical products are resolved before the EMA provides its scientific opinion to the EC given that all Member States are represented in the risk evaluation as part of EMA’s scientific committees. Consequently, it is not the long-term credible commitment for common regulation based on scientific evidence, instead, the Member States’ short-term interests and politics, which ultimately determine the food regulatory framework. Here the second and biggest dissimilarity. We want to emphasize though that it is important that EFSA remains politically independent and autonomous. Our recommendations do not suggest that EFSA should become politicized. Nor do we suggest that EFSA’s scientists should act on behalf of their governments, but rather that representation in EFSA increases the chance that member state being properly and scientifically informed. If an expert from a particular country is member of an EFSA panel, then we believe that the chances increase that the scientific conclusions reach that country’s decision makers in a more direct manner (e.g., through personal communications with that expert) and that this will influence the voting behavior in the PAFF, similar to what happens at EMA. At EFSA, the lack of representation is creating a politicized situation in comitology. With the appropriate representation from all Member States all scientific differences in national opinions would be resolved before EFSA provides its scientific opinion to the Commission, who will then basically approve EFSA’s opinion as it happens with EMA’s opinions.

Moreover, “the regulation of foodstuff mainly has to rely on post-marketing control” because the foodstuff market is much more fragmented with the exception of food additives, as well as novel foods and food ingredients, especially products derived from GMOs, which need to be authorized before they get access to the Single Market^44^ (Krapohl, 2004). In contrast, the specific rules for the relatively homogeneous pharmaceuticals products that are produced by large companies allow the premarket^45^ evaluation and regulation^46^ (Krapohl, 2004), which is another difference in the rules governing the two agencies.

High autonomy and low political influence is what should characterize a regulatory agency. This is relevant for initiatives and collaborations with other regulatory authorities or the industry because the higher the autonomy and the lower the role of politics the more attractive the agency is to collaborate with. However, as explained, the current absence of a long-term credible commitment for common regulation based on scientific evidence is making EFSA a vulnerable target to political interests. If the wish is to have an independent agency able to provide advice based on sound science, several changes have to be made in the organization of the agency.

In the effort to improve citizens and stakeholders confidence in transparency and sustainability of the EU risk assessment, the Commission introduced changes in the legal framework on GFL and recently adopted a new regulation based on Art. 43, 114, and 168 (4) (b) of the Treaty of the Functioning of the European Union. The new regulation emphasizes the need for transparency and sustainability of the EU risk assessment in the food chain. The regulation aims to harmonize the procedures followed in the functioning of EFSA with these followed by other scientific agencies such as European Chemical Agency (ECHA) and EMA, since the governance of EFSA is not in line with the Common Approach on decentralized agencies, such as the composition of the Management Board. The specific changes in the functioning of EFSA introduced by the new regulation are going in the right direction (Box 2) (points 1, 2, and 4). They can contribute to a more open and qualified communication on risks, which can decrease fear by providing clear information about real versus perceived risks. However, there is space for improvement.

**Box 2. The four main elements of the New Regulation agreement aim at:**

• **Ensuring more transparency:** Citizens will have automatic access to all studies and information submitted by industry during the risk assessment process. Stakeholders and the general public will also be consulted on submitted studies. At the same time, the agreement will guarantee confidentiality, in duly justified circumstances, by setting out the type of information that may be considered significantly harmful for commercial interests and therefore cannot be disclosed.
• **Increasing the independence of studies:** The European Food Safety Authority will be notified of all commissioned studies to guarantee that companies applying for authorizations submit all relevant information and do not hold back unfavourable studies. The Authority will also provide general advice to applicants, in particular SMEs, prior to the submission of the dossier. Commission may ask the Authority to commission additional studies for verification purposes and may perform fact-finding missions to verify the compliance of laboratories/studies with standards.
• **Strengthening the governance and the scientific cooperation:** Member States, civil society and European Parliament will be involved in the governance of the Authority by being duly represented in its Management Board. Member States will foster the Authority’s scientific capacity and engage the best independent experts into its work.
• **Developing comprehensive risk communication:** A general plan for risk communication will be adopted and will ensure a coherent risk communication strategy throughout the risk analysis process, combined with open dialogue amongst all interested parties.

Source: http://europa.eu/rapid/press-release_IP-19-1030_en.htm

Point 1 on ensuring more transparency: The access of the public to information related to the risk assessment at early stage while ensuring duly justified confidentiality is significant and also relevant to Point 2 on increasing the independence of studies. However, this change combined with the proposed pre-submission procedure, which can be useful especially for small and medium size companies, would require more financial and human resources by EFSA. Another challenge concerns the way it will be justified what requires confidentiality and what not so that it will not threaten innovation and business property.

Point 3 indicates that the new Regulation introduces changes in the governance of EFSA by increasing the involvement of the Member States in the Management Board, and in the nomination of members of the scientific panels, §14 and §15. However, this change focuses on the Management Board that is involved in the administration of finances but not directly in the risk assessment. Besides, this change might increase the number of available qualified risk assessors but it does not address the representation of the member states scientific divergences at an early stage. Consequently, this change does not allow deliberation on scientific divergences among the member states at an early stage, on scientific basis, as it happens in EMA during the risk evaluation. One of the great challenges is how to ensure scientific clarity. This can only happen by having an extended pool of independent scientific evidence and strong collaboration among most, if not all, Member States and the EFSA, which is relevant to Point 2. The need for available tools to support cooperation among between EFSA and the Member States is emphasized by a significant number of respondents^47^ (40% of citizens and stakeholders).

Point 4 concerns changes in governance. The main changes linked to the risk assessment process are introduced in Art. 25, Art. 28 (5) and Art.32. Nevertheless, these changes can improve the communication with the public about the relevant scientific evidence used in the risk assessment process, scientific evidence still has important role to play for dispelling widespread misconceptions, so the communication should be science-based and more in a form of public debates as previously suggested (Qaim, 2016, p. 115). Therefore, there is a need for improvement and simplification of the communication with the public. Better and simpler information by legitimate actors based on scientific facts and democratic principles can shape public opinion positively, beyond biased information and prejudices. It is important for the public to understand how technology can contribute to food safety, food security, and sustainable agriculture development, hence it needs to be utilized and expanded. Biased information and prejudices distorts public opinion.

Unfortunately, the changes introduced by the Regulation do not generate any significant changes with respect to the risk assessment process and the representation of scientists from all the member states, which is crucial for creating trust in public opinion among the member states people. Instead, the amendments mostly focus on increasing and improving communication and openness of the process. Consequently, it is not clear if the new Regulation is able to overcome the existing backdrops, as the governance processes and organization differences are which determine the Commission’s final authorization decision.

## Author Contributions

SC took the initiative and prepared the early draft. All authors made a substantial, direct and intellectual contribution to the work, and approved it for publication.

## Conflict of Interest

The authors declare that the research was conducted in the absence of any commercial or financial relationships that could be construed as a potential conflict of interest.

## Footnotes

1 Initially known as SANCO.

2 https://www.efsa.europa.eu/sites/default/files/corporate_publications/files/Eurobarometer2019_Food-safety-in-the-EU_Full-report.pdf, Special Eurobarometer Wave EB91.3.

3 https://chemicalwatch.com/15504/efsa-delays-bpa-exposure-assessment, https://www.icis.com/explore/resources/news/2008/12/10/9178492/basf-slams-efsa-delay-on-amflora-safety-decision/

4 http://www.europarl.europa.eu/factsheets/en/sheet/149/european-citizens-initiative

5 http://ec.europa.eu/citizens-initiative/public/initiatives/successful

6 http://ec.europa.eu/transparency/regdoc/rep/3/2017/EN/C-2017-8414-F1-EN-ANNEX-1-PART-1.PDF. The initiative was registered 25/01/2017 and was answered 12/12/2017.

7 http://ec.europa.eu/transparency/regdoc/rep/3/2017/EN/C-2017-8414-F1-EN-MAIN-PART-1.PDF

8 These are GMOs, smoke flavorings, food contact materials, food additives, food enzymes and flavorings, plant protection products and novel foods.

9 http://www.europarl.europa.eu/legislative-train/theme-environment-public-health-and-food-safety/file-transparency-and-sustainability-of-the-eu-risk-assessment-in-the-food-chain

10 http://europa.eu/rapid/press-release_MEMO-18-2942_en.htm

11 https://ec.europa.eu/food/sites/food/files/gfl_transparency_comm_proposal_synopsis_20180410_en.pdf

12 We did not compare EFSA to EEA or ECDC, for example, because these two agencies do not perform risk assessment of products for the market. We did not chose EEA and ECHA because they do not directly provide scientific opinions to DG SANTE as both EFSA and EMA do.

13 https://www.foodnavigator.com/Article/2019/04/17/Europe-s-new-rules-for-food-safety-approval-Building-trust-in-science

14 http://www.arc2020.eu/pioneers-gm-maize-1507-a-case-history/

15 Please see Eriksson et al. (2019) and Lehrman (2014) for a detailed presentation of the authorization process in the comitology procedure.

16 Point suggested by one of the reviewers.

17 https://eur-lex.europa.eu/legal-content/EN/TXT/PDF/?uri=CELEX:32002R0178&from=EN

18 http://www.efsa.europa.eu/en/people/operationalmanagement

19 Panel on Animal Health and Welfare (AHAW), Panel on Biological Hazards (BIOHAZ), Panel on Food Contact Materials, Enzymes and Processing Aids (CEP), Panel on Contaminants in the Food Chain (CONTAM), Panel on Food Additives and Flavorings (FAF, Panel on Additives and Products or Substances used in Animal Feed (FEEDAP), Panel on Genetically Modified Organisms (GMO), Panel on Nutrition, Novel Foods and Food Allergens (NDA), Panel on Plant Health (PLH) and PPR.

20 The latest mandate for the Scientific Committee and the ten panels began on 1 July 2018.

21 http://www.efsa.europa.eu/en/howwework/workingpractices

22 https://ec.europa.eu/food/committees/paff_en

23 For a detailed description of the process (see Lehrman et al., 2014, pp. 69–70), https://www.slu.se/globalassets/ew/org/centrb/mbiot/publikationer/shapingourfood_mistrabiotech_web_140625.pdf.

24 REGULATION (EC) No 726/2004 OF THE EUROPEAN PARLIAMENT AND OF THE COUNCIL of 31 March 2004 laying down Community procedures for the authorization and supervision of medicinal products for human and veterinary use and establishing a European Medicines Agency (Text with EEA relevance) (OJ L 136, 30.4.2004, p. 1).

25 https://www.ema.europa.eu/en/committees/how-committees-work

26 https://www.ema.europa.eu/en/committees/how-committees-work; https://www.ema.europa.eu/documents/other/prac-rules-procedure_en.pdf

27 https://www.ema.europa.eu/human-regulatory/post-authorisation/referral-procedures

28 http://www.greenpeace.org/eu-unit/en/News/2010/first-citizens-initiative/

29 http://europa.eu/rapid/press-release_IP-18-2563_en.htm

30 https://ec.europa.eu/food/sites/food/files/gfl_fitc_executive_summary_2018_en.pdf

31 The Advisory Group on Animal and Plant Health discussed the Commission’s proposal on transparency and sustainability of the EU food and feed safety risk assessment model on 5 February 2018.

32 REGULATION OF THE EUROPEAN PARLIAMENT AND OF THE COUNCIL on the transparency and sustainability of the EU risk assessment in the food chain amending Regulation (EC) No 178/2002 (on general food law), Directive 2001/18/EC (on the deliberate release into the environment of GMOs), Regulation (EC) No 1829/2003 (on GM food and feed), Regulation (EC) No 1831/2003 (on feed additives), Regulation (EC) No 2065/2003 (on smoke flavorings), Regulation (EC) No 1935/2004 (on food contact materials), Regulation (EC) No 1331/2008 (on the common authorization procedure for food additives, food enzymes and food flavorings), Regulation (EC) No 1107/2009 ([on plant protection products) and Regulation (EU) No 2015/2283 (on novel foods) https://ec.europa.eu/transparency/regdoc/rep/1/2018/EN/COM-2018-179-F1-EN-MAIN-PART-1.PDF.

33 Fifteen trade and business associations, four Non-Governmental Organizations (NGOs), one Member State (MS), and one citizen submitted feedback. 471 people participated in the OPC, 318 citizens and 153 stakeholders.

34 https://www.bechbruun.com/en/news/2019/new-eu-food-regulation-may-influence-the-disclosing-of-legitimate-business-information

35 https://www.europarl.europa.eu/doceo/document/A-8-2018-0417-AM-132-140_EN.pdf?redirect

36 https://www.euractiv.com/section/agriculture-food/news/new-food-transparency-rules-risk-falling-victim-to-eu-institution-spat/ and https://www.euractiv.com/section/agriculture-food/news/meps-ready-to-negotiate-efsas-transparency-rule-but-need-to-find-a-new-negotiator/

37 The EP suggested 112 amendments (see amendments at http://www.europarl.europa.eu/doceo/document/A-8-2018-0417_EN.html?redirect).

38 http://www.europarl.europa.eu/RegData/etudes/BRIE/2018/630315/EPRS_BRI(2018)630315_EN.pdf.

39 https://www.consilium.europa.eu/en/press/press-releases/2019/02/12/safe-and-transparent-food-chain-provisional-agreement-on-availability-and-independence-of-scientific-studies/

40 https://ec.europa.eu/food/sites/food/files/gfl_transparency_comm_proposal_20180410_factsheet_en.pdf

41 http://europa.eu/rapid/press-release_MEMO-19-1031_en.htm

42 https://europa.eu/rapid/press-release_MEMO-19-1031_en.htm

43 https://www.efsa.europa.eu/sites/default/files/event/mb180321/mb180321-i1.pdf

44 Sebastian Krapohl, “Credible Commitment in Non-Independent Regulatory Agencies: A Comparative Analysis of the European Agencies for Pharmaceuticals and Foodstuffs,” (2004) *ELJ* 5 518, 519.

45 A post-market control also applies for pharmaceuticals (Pharmacovigiliance), but this works just as a subsequent fire-alarm control.

46 Krapohl, *supra* note 42, 519.

47 https://ec.europa.eu/social/BlobServlet?docId=21019&langId
